# A Phase I Dose-Escalation Clinical Trial to Assess the Safety and Efficacy of Umbilical Cord-Derived Mesenchymal Stromal Cells in Knee Osteoarthritis

**DOI:** 10.1093/stcltm/szad088

**Published:** 2024-02-16

**Authors:** Jose Matas, Cynthia García, Daniela Poblete, Rolando Vernal, Alexander Ortloff, Noymar Luque-Campos, Yessia Hidalgo, Jimena Cuenca, Catalina Infante, Maria Ignacia Cadiz, Maroun Khoury, Patricia Luz-Crawford, Francisco Espinoza

**Affiliations:** Centro de Terapia Celular, Clínica Universidad de los Andes, Santiago, Chile; IMPACT, Center of Interventional Medicine for Precision and Advanced Cellular Therapy, Santiago, Chile; Centro de Investigación e Innovación Biomédica, Facultad de Medicina, Universidad de Los Andes, Santiago, Chile; Periodontal Biology Laboratory, Facultad de Odontología, Universidad de Chile, Santiago, Chile; Periodontal Biology Laboratory, Facultad de Odontología, Universidad de Chile, Santiago, Chile; Departamento de Ciencias Veterinarias y Salud Pública, Facultad de Recursos Naturales, Universidad Católica de Temuco, Temuco, Chile; IMPACT, Center of Interventional Medicine for Precision and Advanced Cellular Therapy, Santiago, Chile; Centro de Investigación e Innovación Biomédica, Facultad de Medicina, Universidad de Los Andes, Santiago, Chile; IMPACT, Center of Interventional Medicine for Precision and Advanced Cellular Therapy, Santiago, Chile; Centro de Investigación e Innovación Biomédica, Facultad de Medicina, Universidad de Los Andes, Santiago, Chile; Cells for Cells and Regenero The Chilean Consortium for Regenerative Medicine, Santiago, Chile; IMPACT, Center of Interventional Medicine for Precision and Advanced Cellular Therapy, Santiago, Chile; Centro de Investigación e Innovación Biomédica, Facultad de Medicina, Universidad de Los Andes, Santiago, Chile; Cells for Cells and Regenero The Chilean Consortium for Regenerative Medicine, Santiago, Chile; Centro de Terapia Celular, Clínica Universidad de los Andes, Santiago, Chile; Centro de Investigación e Innovación Biomédica, Facultad de Medicina, Universidad de Los Andes, Santiago, Chile; Cells for Cells and Regenero The Chilean Consortium for Regenerative Medicine, Santiago, Chile; Centro de Investigación e Innovación Biomédica, Facultad de Medicina, Universidad de Los Andes, Santiago, Chile; Cells for Cells and Regenero The Chilean Consortium for Regenerative Medicine, Santiago, Chile; IMPACT, Center of Interventional Medicine for Precision and Advanced Cellular Therapy, Santiago, Chile; Centro de Investigación e Innovación Biomédica, Facultad de Medicina, Universidad de Los Andes, Santiago, Chile; Cells for Cells and Regenero The Chilean Consortium for Regenerative Medicine, Santiago, Chile; IMPACT, Center of Interventional Medicine for Precision and Advanced Cellular Therapy, Santiago, Chile; Centro de Investigación e Innovación Biomédica, Facultad de Medicina, Universidad de Los Andes, Santiago, Chile; Centro de Terapia Celular, Clínica Universidad de los Andes, Santiago, Chile; IMPACT, Center of Interventional Medicine for Precision and Advanced Cellular Therapy, Santiago, Chile; Centro de Investigación e Innovación Biomédica, Facultad de Medicina, Universidad de Los Andes, Santiago, Chile; Cells for Cells and Regenero The Chilean Consortium for Regenerative Medicine, Santiago, Chile

**Keywords:** umbilical-cord-derived mesenchymal stromal cells, osteoarthritis, dose escalation, murine OA model, phase I clinical trial

## Abstract

Osteoarthritis (OA) is the most common degenerative joint disease. Mesenchymal stromal cells (MSC) are promising cell-based therapy for OA. However, there is still a need for additional randomized, dose-dependent studies to determine the optimal dose and tissue source of MSC for improved clinical outcomes. Here, we performed a dose-dependant evaluation of umbilical cord (UC)-derived MSC (Celllistem) in a murine model and in knee OA patients. For the preclinical study, a classical dose (200.000 cells) and a lower dose (50.000 cells) of Cellistem were intra-articularly injected into the mice knee joints. The results showed a dose efficacy response effect of Cellistem associated with a decreased inflammatory and degenerative response according to the Pritzker OARSI score. Following the same approach, the dose-escalation phase I clinical trial design included 3 sequential cohorts: low-dose group (2 × 10^6^ cells), medium-dose group (20 × 10^6^), and high-dose group (80 × 10^6^). All the doses were safe, and no serious adverse events were reported. Nonetheless, 100% of the patients injected with the high-dose experienced injection-related swelling in the knee joint. According to WOMAC total outcomes, patients treated with all doses reported significant improvements in pain and function compared with baseline after 3 and 6 months. However, the improvements were higher in patients treated with both medium and low dose as compared to high dose. Therefore, our data demonstrate that the intra-articular injection of different doses of Cellistem is both safe and efficient, making it an interesting therapeutic alternative to treat mild and symptomatic knee OA patients.

Trial registration ClinicalTrials.gov NCT03810521.

## Graphical Abstract

**Graphical Abstract:**
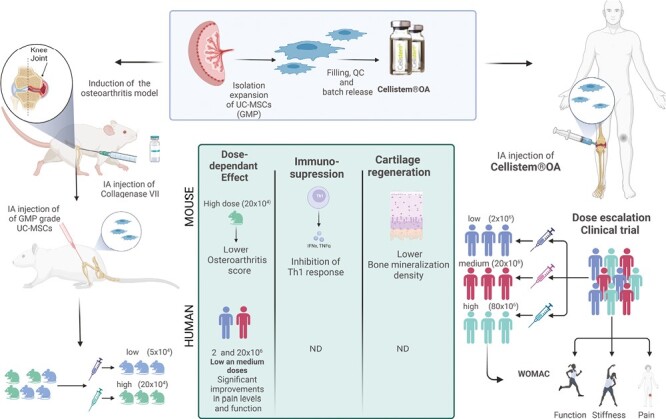

**Lessons Learned**

- Low and middle doses were more efficient in OA patients.
- The infusion of a high Cellistem dose induces local swelling associated to inflammation.
- Is critical to perform human dose escalation studies to validate the pre-clinical outcomes.

**Significance Statement**

This is the first dose escalation clinical study that evaluated the therapeutic efficacy of UC-derived MSC (Cellistem) for moderate and symptomatic knee OA. The study included a preclinical model and a phase I clinical trial study with 3 Cellistem doses. Our results showed that in vivo murine model of OA display a dose-dependent effect according to histological analysis. On the other hand, all injected doses in the clinical trial were safe and displayed significant inhibition of pain and inflammation according to WOMAC. Therefore, Cellistem demonstrated a clinical therapeutic effect in OA even when used at lower dose.

## Introduction

Osteoarthritis (OA) is the most frequent degenerative joint disease worldwide with a continuously increasing prevalence due to the gradual aging of the world population. It is characterized by the progressive loss of articular cartilage, causing chronic pain, inflammation, and increasing disability, ultimately associated with the total loss of joint function.^1^ Unfortunately, OA’s current therapy comprises symptomatic pain treatment without preventing other degenerative processes.^2^ According to this, cell-based therapy with mesenchymal stromal cells (MSCs) arises as an attractive therapeutic tool to treat OA due to their anti-inflammatory and chondrogenic properties.^3^ They can be isolated from several adult tissues including adipose tissue (AD), menstrual blood, bone marrow (BM), and umbilical cord (UC).^4-6^ Currently, several preclinical studies and clinical trials have been performed using autologous or allogenic MSC mainly from AD, UC, and BM. These have reported their safety and efficacy in preclinical murine models and in patients with OA using a single dose.^7-13^ As we have previously reported,UC-MSC offer several advantages over other MSC sources (including BM and MB). UC-MSC outplace other tissue origins in terms of yield, differentiation potential, and immunosuppresive capacities.^4,14^ Hence, we focused our efforts in evaluating the therapeutic application of UC-MSC in OA. Indeed, in our previous controlled randomized phase I/II trial, we described the safety and anti-inflammatory effect of the intra-articular injection of umbilical-cord-derived MSC (Cellistem) with a dose of 20 × 10^6^ cells (comparable to the medium dose used in the current study).^10^ Due to the variability of cell doses described in the literature and the considering regulatory and clinical scalability, it is critical to conduct a dosage range study to determine the optimal Cellistem dose for treating knee-OA.^15,16^ Nonetheless, this study was not aimed to determine if single or repetitive doses display a superior therapeutic effect, but rather to see whether patients treated with UC-MSC will exhibit a dose-response impact on the course of the disease. Hence, in the present study, we used an in vivo experimental mouse model of OA to demonstrate the preclinical therapeutic impact of two distinct dosages of UC-derived MSC (Celllistem) and the evaluation of the safety and efficacy of a dose-escalation protocol of intra-articular injected Celllistem in patients with mild and symptomatic knee OA following the regulatory agency approval.

## Materials and Methods

### Manufacturing of Clinical-Grade Cellular Product

The umbilical cord-derived mesenchymal stromal cells (UC‐MSCs), labelled as Cellistem, Cells for Cells, Chile, were isolated and characterized as previously described to obtain a high-quality product for clinical use.^10,17^ Characterization criteria was according to the International Society for Cellular Therapy^18^ and included tri differentiation capacities, specific surface markers expression, immunosuppressive capacities, thrombospondin 2 production, and Karyotype analysis. Cells were used and characterized at passage 5.

The release criteria included the absence of macroscopic clumps, cell number, sterility (mycoplasma, aerobic and anaerobic hemocultures, and Gram stain), endotoxin (≤0.5 EU/mL); and a viability > 80%, with an identity and purity pattern characterized by ≥ 95% positivity for CD73, CD90, and CD105, and negativity (≤2%) for the expression of CD45, CD34, CD14, and Human Leukocyte Antigen‐DR isotype (HLA‐DR). Cells (2 × 10^6^, 20 × 10^6^, and 80 × 10^6^) were suspended in a final volume of 3 mL (saline solution, 5% AB^+^ human plasma) and dispensed in masked 5‐mL syringes to treat individual patients accordingly with the study design.

### Collagenase-Induced Osteoarthritis Model

Collagenase-induced OA (CIOA) model was carried out as previously described^5^ and according to the guidelines and regulations of the Ethical Committee for animal experimentation from the Universidad de los Andes Approval CEC201939. Briefly, 1U type VII collagenase in 5 µL saline was intra-articular (IA) administered in the knee joint of C57BL/6 mice (10 weeks old) at days 0 and 2. Groups of 10 mice received an IA injection of UC-MSC (2 × 10^5^—high dose and 0.5 cells × 10^5^—low dose/5 µL saline), on days 7 and 14. On day 42, mice were euthanatized and paws were carefully dissected to remove smooth tissues for micro-CT scanner and then for fixation in 4% formaldehyde for histological analysis.

### MicroCT Analysis

The samples were analyzed using X-ray microtomography, Micro-CT SkyScan 1278 (Bruker, Belgium, 0.5 mm aluminum filter, 20-65 kV, 500 µA, resolution of 50 µm, 0.5° rotation angle), under characteristics defined by the equipment operator. 3D scans were reconstructed using NRecon software (Bruker, Belgium). Misalignment compensation, ring artifacts and beam-hardening were configurated to obtain a correct reconstruction of each paw. Bone mineral density was quantified in 4 knee zones: lateral subchondral, medial subchondral, lateral femur, and medial femur of each paw (CTAn Software, Bruker, Belgium).

### Histological Analysis

Hind paws were decalcified after a 2-week incubation within a formic acid 5% solution and then embedded in paraffin. Tibias were sectioned frontally as previously described^19^ and stained with safranin O fast green staining. Quantification of the degradation of cartilage was performed using the modified Pritzker OARSI score as described.^5,20^

### Biodistribution Analysis

After reaching 80% confluence, Cellistem was trypsinized and stained with DiR (DiIC_18_^7^; 1,1ʹ-dioctadecyl-3,3,3ʹ,3ʹ-tetramethylindotricarbocyanine iodide) (Biotium) at 10 µM for 20 minutes at 37 °C. Detection of fluorescent imaging of OA mice intraarticular injected with DiR-Cellistem (2 × 10^5^ cells/5 µL) into the right knee joint and the contralateral knee was used as a sodium chloride sham control. Mice were followed for 7 days post-injection of Cellistem by performing staining visualization using the Odyssey CLx Imager (LI-COR) for 1 h, 72 and 7 days post Cellistem infusion with the Mouse Pad accessory to maintain the body temperature of anesthetized mice at 37 °C.

### Immunogenic Analysis In Vivo

Mice were euthanatized on day 14 of OA induction and the drain popliteal lymph nodes were recovered for disaggregation. Extracted cells were passed through a 40-μm filter (cell strainer; BD Falcon) and centrifuged at 1680 rpm for 6 minutes. Then, cells were cultured with PMA (50 ng/mL) (Sigma) and Ionomycin (1 µg/mL) (Sigma-Aldrich) in the presence of 10 μg/mL brefeldin A (eBiosciences). After 4 hours, standard intracellular staining was carried out to identify the CD4+, IFN-γ+, IL17+, CD25 + high, and Foxp3 + cells. For this, cells were fixed and permeabilized using the Cytofix/Cytoperm kit (BD Biosciences), according to the manufacturer’s instructions. The acquisition was performed with a FACS Canto II using the FlowJo software (versión 10.0.7) measured by flow cytometry.

### Study Design

A dose-response clinical trial aiming the safety and efficacy of three different doses of an intra-articular knee injection of Cellistem was planned. The study was registered at ClinicalTrials.gov (NCT02580695) and approved by the local Ethics Committee of Universidad de los Andes (CEC201861). The protocol was conducted under good clinical practice guidelines and the declaration of Helsinki.

### Patients

Participants were recruited between March and May 2019 at the University of Los Andes Clinical Center in Santiago, Chile. Patients were included in the study based on the following criteria: age between 30 and 75 years, symptomatic knee OA (defined by daily pain at the affected joint for at least 3 months before inclusion and visual analog scale equal or superior to 40 mm), grades 1-3 Kellgren-Lawrence radiographic changes. Patients were excluded if they had one of the following conditions: meniscal rupture, bilateral symptomatic knee-OA, significant axial deviation defined by valgus (>10°) or varus (>5°) deformity, disease of the hip and/or spine, local or systemic infection, any form of secondary arthritis, previous malignancy, intra-articular injection in the affected knee with steroids or hyaluronic acid in the past 6 months. All randomized patients provided written informed consent.

### Intervention

Sixty individuals were screened and forty of them were finally recruited among one of the following groups: High-dose (HD) Cellistem (injection of 80 × 10^6^ UC-MSCs), medium-dose (MD) Cellistem (injection of 20 × 10^6^ UC-MSCs) and low-dose (LD) Cellistem (injection of 2 × 10^6^ UC-MSCs). Intra-articular injection contained MSCs diluted in 3cc of saline with 5% AB plasma. All injections were identical. In the HD Cellistem group, recruitment was stopped early due to an interim analysis showing a higher frequency of adverse events after injection. For this reason, HD has half of the patients (*n* = 8) than both MD and LD groups (*n* = 16). The final allocation ratio was 1:2:2 (HD:MD:LD).

### Outcomes

The primary endpoint was safety, according to the frequency of treatment-related adverse events in each group. The secondary endpoint was efficacy. These outcomes were assessed using the following tools: pain visual analog scale (VAS), Western Ontario and Mc Master Universities Arthritis Index (WOMAC) Spanish validated version and Whole-Organ Magnetic Resonance Imaging Score (WORMS) for knee osteoarthritis through a 1.5T MRI. Blinded readings were performed independently by two specialized radiologists.

### Procedures and Follow-up

All injections were performed by the same orthopedic surgeon who was blinded to the dose administered. Patients were indicated to avoid physical activity for 5 days after the procedure. A register of analgesics used by the patient after infiltration was recorded during the first week. Clinical outcomes were evaluated at 1, 4, 12, and 24 weeks by an independent staff, blinded to treatment and not related to patient care. (See Table 4 for flow chart)

### Statistical Analysis

For the preclinical assay, results were expressed as the mean ± SD. For the in vivo studies (CIOA), 8 to 10 animals were used for each experimental or control group, and experiments were repeated at least two independent times. The *P*-values were generated by parametric analysis using the one-way ANOVA test for multiple comparisons. *P* < .05 (*), *P* < .01 (**), or *P* < .001 (***) was considered statistically significant. All the analyses were performed using the GraphPad Prism TM 6 software (GraphPad Software, San Diego, CA, USA).

For the clinical trial, the sample description included the frequencies of each category for qualitative variables and mean plus SD for quantitative variables. A Kruskal-Wallis one-way analysis-of-variance-by-ranks test was used to examine whether differences in quantitative variables were significant among groups at baseline and during follow‐up. The significance level was set at 5% for all tests. All statistical analyses were performed using the R platform (v3.4.1; R Development Core Team) in adherence to Good Statistical Practice in Clinical Research.

## Results

### UC‐MSC Batch Selection and Characterization

UC‐MSC batches were evaluated according to the expression of different mesodermal (CD73, CD90, CD105) a non-mesodermal markers (CD45, CD34, CD11b, CD19, and HLA-DR), the tri‐differentiation potential to mesodermal lineages and the immunosuppressive abilities to compliance the minimum criteria of the International Society for Cellular Therapy.^18^ For that purpose, UC-MSC were thawed between passages 3-4 and immunophenotypic characterization was performed by flow cytometry (Supplementary Fig.S1A). Differentiation potential was determined by culturing the cells under specific culture conditions to induce the differentiation into chondrocytes, adipocytes or osteoblast. For this, UC-MSCs were stained to assess the adipogenic (Oil Red O), osteogenic (Alizarin Red), and chondrogenic (Safranin O) differentiation (Supplementary Fig. S1B). Finally, to determine the immunosuppressive abilities of UC-MSC, peripheral blood mononuclear cells (PBMC) were isolated from healthy donors, activated with phytohemagglutinin (PHA), and cultured in the presence or absence of UC-MSC. After 3 days of co-culture, proliferation and the generation of anti-inflammatory Treg cells were evaluated by FACS (Supplementary Fig. S1C). Our results demonstrated that the cells selected for the preclinical and clinical trial meet the ISCT criteria since they showed the classical MSC immunophenotype. Indeed, cells showed more than 95% of positive stain for mesodermal markers such as CD90, CD73, and CD105 while showing negative expression (less than 5%) of non-mesodermal antigens ( Supplementary Fig. S1A). Moreover, they were also able to differentiate into adipocytes, chondrocytes, and osteoblast as demonstrated by positive staining for Oil Red O, alizarin red, and safranine O, respectively (Supplementary Fig. S1B). Finally, the immunosuppressive abilities of Cellistem were shown by their capacity to inhibit the proliferation of T-CD4 cells while inducing the generation of Treg cells (CD4 + CD25 + FOXP3+) (1C). In terms of paracrine factors, it has been previously described that the production of thrombospondin‐2 (TSP2) is a key chondrogenic and chondroprotective factor.^21^ Therefore, we evaluated the secretion of TSP2 in 3 different UC-MSC donors isolated under GMP conditions. Accordingly, we selected the UC-MSC source with higher TSP‐2 secretion as an internal potency test (Supplementary Fig. S1D). Finally, a karyotype analysis was performed to evaluate the potential genetic abnormalities of the cells. Our results revealed no clonal abnormalities (Supplementary Fig. S1E). Moreover, the batch selected demonstrated no tumorigenic activity when they were injected into SCID mice (data not shown). Altogether these data allowed us to qualify the different batches of UC-MSC isolated under GMP conditions and to select the UC-MSC source with the higher score of phenotype, function, and TSP-2 secretion to become our product Cellistem for this preclinical and clinical dose-response trial.

### Cellistem Displays a Dose-Dependent Anti-Osteoarthritic Effect in a Murine Model of OA

It has been well described that MSC protects chondrocytes from degeneration associated with OA, protecting mice from OA development.^5,19,20,22^ Therefore, since MSC possesses an intrinsic ability to regenerate articular cartilage,^23^ we aimed to determine the optimal dose of Cellistem that would result in the best possible outcome, as determined by chondrocyte protection in the CIOA murine model.

**Figure 1.**
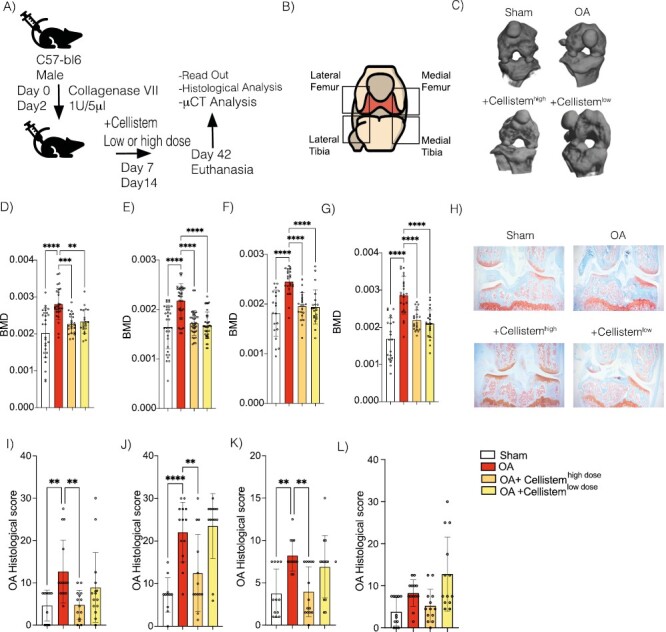
Preclinical dose response Cellistem efficacy evaluation in the murine collagenase-induced osteoarthritis model. (**A**) Experimental design of the dose response preclinical trial in the CIOA murine model. (**B**) Representative figure showing the different knee areas evaluated for microCT and histological analysis. (**C**) Representative 3D images of XY axes photography selection evaluated by MicroCT analysis. Bone mineral density average analyses of the (**D**) medial tibia, (**E**) lateral tibia, (**F**) medial femur, and (**G**) lateral femur. (**H**) Representative histological images of CIO mice not treated (collagenase) or treated with different doses of Cellistem. Histological OA score analyses of the (**I**) Medial Tibia, (**J**) Lateral Tibia, (**K**) Medial Femur, and (**L**) Lateral Femur. Results are expressed as bone mineral density (mm^3^), a histomorphometry analysis of 3D images of articular cartilages and as OA score of histological sections of knee joints of the mice (*n* = at least 15/group in 3 independent experiments). Results are expressed as the mean ± SD; *P ≤ .05, **P < .01, ***P < .001 (one-way ANOVA-test).

Thus, we evaluated in vivo the effect of intra-articular (IA) injection of different doses of Cellistem (50.000 and 200.000) in CIOA mice. These doses were selected according to previously published data showing therapeutic efficacy of the selected dose^24^ and the highest concentration of cells that can be packed in the pre-determined injection volume. When the bone mineral density (BMD) changes were analyzed using micro-CT, the 4 knee zones treated with both Cellistem doses showed significant changes in bone degeneration compared to OA control mice (Fig. 1D-1G). No significant differences were observed between doses of Cellistem (Fig. 1E and 1G). Conversely, histological analysis showed that the OA score was significantly lower in the medial and lateral tibia (mean histological score of 4.5 for high Cellistem dose vs. 12.5 for OA mice in medial tibia and 12.5 for high Cellistem dose vs. 23 for OA mice in lateral tibia) and in medial femur (4 for high Cellistem dose vs. 8 for OA mice; Fig. 1H-1L). No differences were observed in the OA score between mice treated with low doses of Cellistem and the untreated mice, used as control (Fig. 1J-1L).

### In Vivo Immunogenic and Biodistribution Analysis of Cellistem

**Figure 2.**
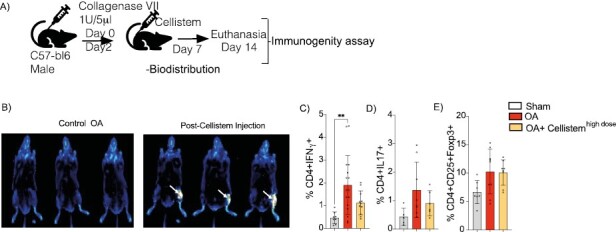
Biodistribution and immunogenic analysis in vivo of Cellistem. (**A**) Experimental design of the biodistribution and immunogenic assay in the CIOA murine model. (**B**) Representative mice images following intra-articular injections with DiR-Cellistem high dose in OA mice (white arrows), evaluated after 0, 7, and 14 days post-treatment by Odyssey CLx Imager. Sodium chloride (NaCl) was used in control OA mice (left images). (**C**) The percentage of proinflammatory and antiinflamatory T-CD4 cells was analyzed in freshly isolated drained popliteal lymph node was evaluated by FACS analysis. Results represent mean ± SD; *P ≤ .05, **P < .01, ***P < .001. One-way ANOVA test of *N* = 10 for 2 independent experiments.

To assess the potential leakage and persistence of the injection, the cells were labeled with DIR before the injection of 200.000 Cellistem in mice. Biodistribution analysis revealed that UC-MSC mostly remain at the site of injection after 7 days post-injection as observed in Fig. 2B. Since one of the main symptoms of OA patients is the inflammation of the joint, we evaluate the immunosuppresive role of Cellistem on the treated joint. For that purpose, after 7 days of the intraarticular injection of Cellistem, mice were euthanaized and the immunosuppresive effect of Cellistem over several proinflammatory and anti-inflammatory T-cells populations was evaluated in the nearby popliteal lymph nodes by FACS. Our results showed that Cellistem significantly inhibits the generation of chronic inflammation associated with proinflammatory Th1 response (Fig. 2C-2E). No differences were observed in terms of proinflammatory Th17 cells nor on the generation of anti-inflammatory Treg cells. Overall, these results indicate that Cellistem displays a dose-response therapeutic efficacy in the CIOA mice that was associated with an inhibition of the pro-inflammatory Th1 response.

### Baseline Characteristics

For the phase I dose-escalation clinical trial, patients were allocated into 3 different doses of Cellistem. A low-dose group (LD) (2 × 10^6^), a medium-dose group (MD) (20 × 10^6^), and a high-dose group (HD) (80 × 10^6^) (Fig. 4). In terms of clinical and structural characteristics at baseline, we did not find any significant difference as shown in Table 1.

**Table 1.** Changes in MRI (WORMS) after 6 months of follow-up.

	Baseline	6 months	*P*-value
LD group	47.8 ± 17.1	49.1 ± 21.1	.88
MD group	39.4 ± 12.2	46.8 ± 15.2	.84
HD group	44.3 ± 14.7	41.8 ± 9.7	.95

**Figure 3.**
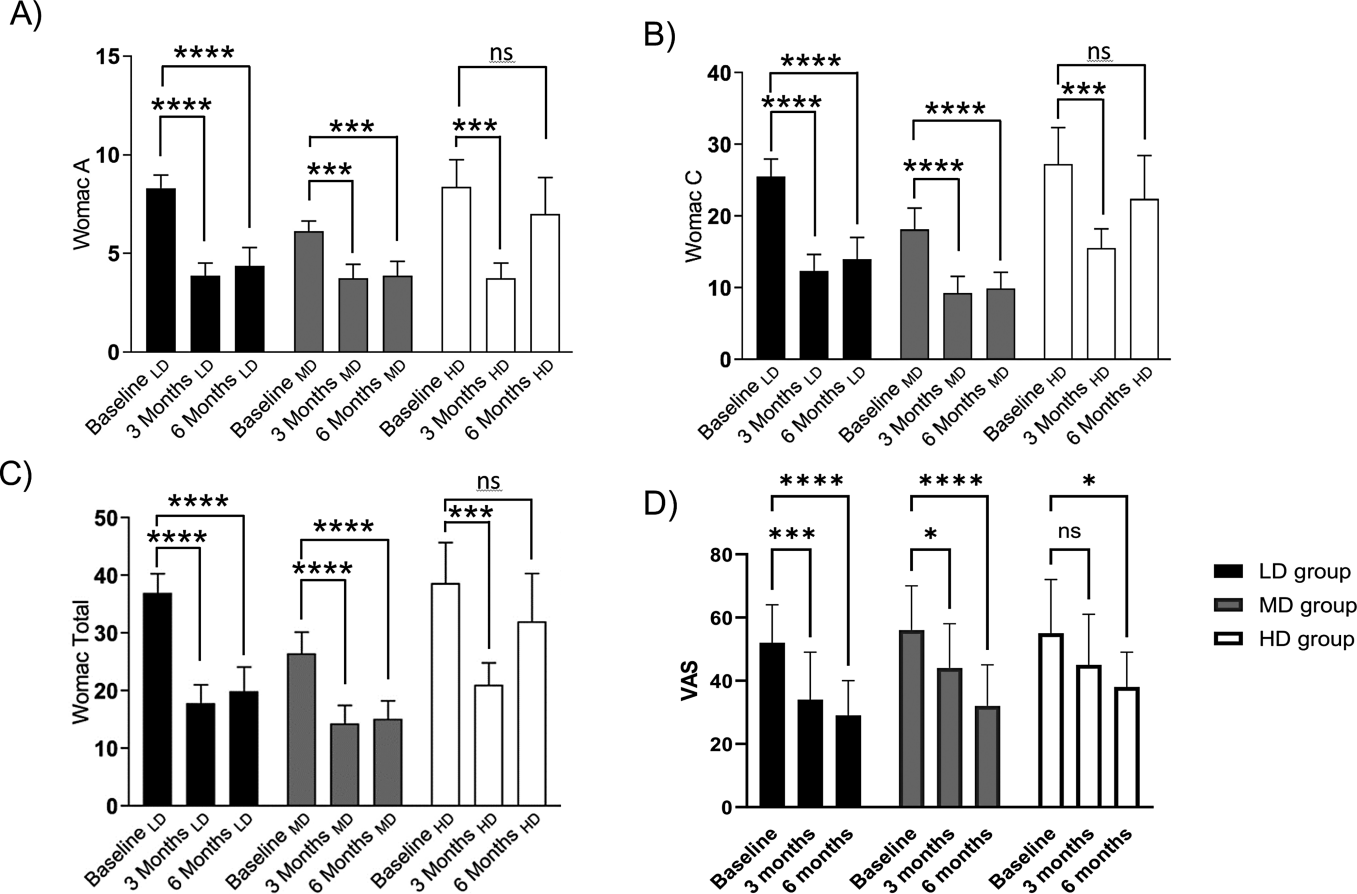
Efficacy clinical outcomes. (**A**) WOMAC‐A pain subscale. (**B**) WOMAC‐C function subscale. (**C**) Total WOMAC. (**D**) VAS analysis. Abbreviations: LD, low dose (2 × 10^6^ UC-MSC); MD, medium dose (20 × 10^6^ UC-MSC); HD, high dose (80 × 10^6^ UC-MSC). WOMAC, Western Ontario and Mc Master Universities Arthritis Index. VAS, Visual Analogue Scale. Results are presented as mean ± SD and were performed to baseline in each group. *P ≤ .05, **P < .01, ***P < .001.

**Figure 4.**
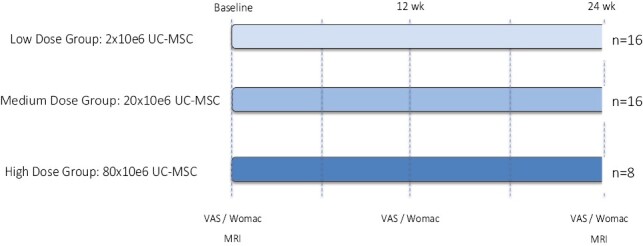
Flow chart of the clinical trial.

### Safety

Our results showed no cases of septic arthritis, disability, neoplasia, or hospitalizations during follow-up. The most part of AEs were occurred follow intra-articular infiltration of cell product and are summarized in Table 2. Detailed AEs case by case are detailed in Supplementary Table 1. Of all AEs registered due to injection, the most common was pain. Duration and intensity of pain were directly correlated with MSC dose. While almost 40% of patients in the LD group experienced clinically significant pain (VAS superior to 40 mm lasting more than 72 hours after infiltration), 100% of HD group patients reported it. Notably, patients receiving the lower MSC dose have less and briefer pain (VAS 4.1) than the other experimental groups. This finding was endorsed by analgesics consumption in the LD group (31% vs over 80% in the other study groups). Additionally, a significant proportion of patients in HD group (37.5%), experienced joint effusion lasting ~1 week after injection. Regarding structural surveillance, no safety signals were reported in MRI analysis at 6 months of follow-up.

**Table 2.** Safety data at 6 months of follow-up.

	LD group *n* = 16	MD group *n* = 16	HD group *n* = 8
Injection-related AE			
Synovitis, *n* (%)	0	1 (6,2)	3 (37.5)
Clinically significant pain*, *n* (%)	6 (37,5)	11 (68,7)	8 (100)
Infection, *n* (%)	0	0	0
Fever, *n* (%)	1 (6,2)	0	0

Data are presented as *n*.

Abbreviation: AE, adverse events.

### Clinical Efficacy Profile

Efficacy endpoints were assessed by measuring WOMAC and VAS (Fig. 3). At 6 months, both LD and MD groups displayed a significantly lower pain and disability compared to baseline. Comparison of groups at the end of follow-up reveals no significant differences between them. WORMS score did not show any significant change in cartilage or any other main descriptor as shown in Table 3.

**Table 3.** Clinical and radiological baseline measures.

	LD group *n = 16*	MD group *n = 16*	HD group *n = 8*	*P*-value
Age, year	52.6 ± 9.9	54.8 ± 12	57.7 ± 13.9	.87
Female, *n* (%)	9 (56)	9 (56)	4 (50)	.94
BMI (kg/m^2^)	28.6 ± 3.8	26.1 ± 3.7	33 ± 4.9	.78
WOMAC, mean (SEM)				
Total	36.9 ± 13.3	26.4 ± 14.8	38.6 ± 20	.22
A—pain	8.3 ± 2.7	6.1 ± 2.1	8.3 ± 3.9	.84
B—Stifness	3.1 ± 1.5	2.1 ± 1.7	3 ± 1.9	0.91
C—Function	25.5 ± 9.9	18.1 ± 12	27.2 ± 14.4	0.75
Kellgren Lawrence (%)				
Grade II	67%	62%	69%	0.88
Grade III	33%	38%	31%	0.91
Knee MRI—WORMS				
Frequency of Involvement				
Cartilage	94%	92%	91%	0.97
Osteophytes	88%	95%	93%	0.96
Menisci	74%	69%	72%	0.94
Score (mean, ±)	47.8 ± 17.1	39.4 ± 12.2	44.3 ± 14.7	0.86

Data are presented as n (%) or mean ± SD. Abbreviations: ^1^LD, low-dose (2 × 10^6^ UC-MSC); ^2^MD, medium-dose (20 × 10^6^ UC-MSC); ^3^HD, high-dose (80 × 10^6^ UC-MSC); ^4^BMI, body mass index; ^5^WOMAC, Western Ontario and Mc Master Universities Arthritis Index; ^6^ MRI, magnetic resonance imaging; ^7^ WORMS Whole-Organ Magnetic Resonance Imaging Score, ^8^SD, standard deviation.

## Discussion

In this study, we performed a dose escalation therapeutic efficacy evaluation of UC-derived MSC (Cellistem) in a murine collagenase induce OA (CIOA) model and a dose escalation non-blind clinical trial for moderate and symptomatic OA treatment. The CIOA model is a mouse model used to evaluate the pathological characteristics of loss of articular cartilage, inflammation and osteophyte formation, features that are also observed in human OA.^25^ The injection of collagenase directly into the cavity of the articular joint shows high reproducibility and generates a relatively homogeneous degree of pathological state that might induce significant amount of inflammation and the same chronic degradation of the subchondral knee as compared to human OA.^26-28^ In the preclinical model, we demonstrated that Cellistem exerts a dose-dependent cartilage protective effect in the collagenase-induced OA model according to histological score.

The therapeutic efficacy of MSC in OA in preclinical studies has been previously reported in different animal OA-models such as murine, rat, and dog.^3,29-31^ Among the MSC sources, BM-MSC, AD-MSC, and UC-MSC are the most used MSC sources for OA treatment.^32^ In our case, we have been focusing our attention on the use of UC-MSC to develop a product with clinical grade denominated Cellistem since UC-MSC are easy to obtain and exhibits a greater immunological and regenerative capacity as compared to other MSC sources.^4^ Indeed, it has been observed that UC-MSCs improve cartilage regeneration and the inflammatory response in rats and rabbits with OA.^31,33^ In the present study, our murine CIOA model showed the preventive role of Cellistem on OA progression that significantly depends on the dose. Indeed, our data showed that 200.000 cells (highest dose in our experimental context) display a better beneficial effect as compared to a lower dose. Moreover, we demonstrated that Cellistem injected at a high dose significantly decreased the percentage of Th1 and Th17 lymphocyte in the popliteal nodes of OA mice. These results showed the anti-inflammatory effect of Cellistem that were associated with an improvement on OA progression. Although the CIOA mice model has many histological characteristics and anatomical features closer to human OA. Certain aspects must be considered before extending these claims to a clinical setting. This is mainly due to the fact that the response and clinical outcome to different dosages may differ between mice and patients, perhaps leading to contradictions in the trial endpoints. For example, human OA has (1) distinct superficial, transitional, radial, and deep zones of chondrocytes; (2) superficial and deep chondrocyte zones thinner than transitional and radial zones, that in mice can be distinctive.^34^ Accordingly, previous work has already demonstrated that the high MSC dose used in the preclinical model display a significant beneficial effect in the progression of the murine OA model without secondary inflammation associated to the quantity of cells.^5,24,30,35^ This therapeutic effect was significantly reduced when the MSC dose was reduced to 1/4 of the original amount.

Regarding the clinical effectiveness and safety of UC-MSC for the treatment of OA, in addition to our study,^10^ few other publications have evaluated the clinical effect of UC-MSC in OA with patients. These studies have injected different UC-MSC doses ranging between 1 × 10^8^ to 1 × 10^7^ millions. In general, all the doses displayed anti-osteoarthritic activity, including reducing pain WOMAC and function.^36-43^ However, Günay et al. observed that after the injection of 1 × 10^8^ cells, 3 patients showed mild effusions that could be related to a potential reaction to the high number of cells.^38^ In line with this study, we observed that 100% of our high-dose patients (8 × 10^7^ M) experience high levels of pain with almost 40% of patients that present some synovitis, therefore we did not continue with the recruitment of patients for this dose. However, with the MD and LD, we observed a significant reduction of WOMAC pain and function corroborating the results previously observed in the other clinical trials. Consequently, these results demonstrated the relevance of the used dose and propose that lower doses might exert their beneficial effect over OA patients probably since a high dose also generate inflammation. After 6 months, the pain level and quality of life of all patients have been significantly improved, in the MD and LD groups. In line with our results, Pers et al. demonstrated that patients treated with low-dose ASCs display the highest significant improvements in pain levels and function as compared with baseline.^9^ Similar results were obtained by Sadri et al., where they also observed that the beneficial effect of ASC was associated with an anti-inflammatory response.^44^ Moreover, we demonstrated that UC-MSC injection is safe, and our results showed that low doses display lower initial pain with high clinical positive results as compared to baseline, at 6 months follow-up. Additionally, our previous study described a controlled randomized phase I/II to treat knee OA with our product Cellistem, observing no severe adverse events and a significant reduction of pain and function compared to baseline, at 1-year follow-up.^10^

To the best of our knowledge, this is the first clinical study to evaluate the dose escalation effect of UC‐MSCs in knee OA, including 6 months follow-up clinical study. Altogether, our results confirm the preclinical and clinical therapeutic efficacy of UC-MSC, their safety and highlight the relevance of the dose used. Indeed, local injection of a low and middle dose of allogeneic Cellistem in mild knee OA patients was safe and displayed a significant inhibition of pain and inflammation according to the WOMAC clinical score. These data also highlight the relevance to perform human dose escalation studies, since the MSC dose-response effect observed on the CIOA murine model did not correlate with the observed outcomes in patients.

## Conclusion

Our results demonstrated that intra-articular administration of Cellistem is safe and that the administration of the optimal dose is critical to diminish AD’s effect and the efficacy outcomes for knee OA treatment. Moreover, despite the low number of patient per experimental group our results demonstrated the therapeutic efficacy of the low dose of Cellistem for OA treatment. However, this study displays significant limitations starting by limited patient number requiring the confirmation of the therapeutic efficacy of the low dose of Cellistem in a larger clinical trial. Moreover,is critical to include a control group to confirmate the positive symptomatic outcome associated to Cellistem treatment. In the following studies, a more accurate cartilage quantification and synovial inflammatory analysis will be performed based on automated analysis of high-resolution MRIs.

## Supplementary Material

szad088_suppl_Supplementary_Figure_1

szad088_suppl_Supplementary_Table_1

## Data Availability

The data that support the findings of this study are available in the methods of this article. Further information regarding the experimental design or the results obtained in this article are available on request from the corresponding author.
