# Supplementary material for: A Phase I Dose-Escalation Clinical Trial to Assess the Safety and Efficacy of Umbilical Cord-Derived Mesenchymal Stromal Cells in Knee Osteoarthritis
Source: Stem Cells Transl Med. 2024 Feb 16;13(3):193–203. doi: 10.1093/stcltm/szad088 (PMC10940813; doi:10.1093/stcltm/szad088)
Supplement: szad088_suppl_Supplementary_Figure_1 [file szad088_suppl_supplementary_figure_1.zip › newfolder/Supp. Figure 1 caption.docx]

**Supplementary Figures**

**Supp.Figure 1. Characterization and selection of Cellistem^TM^** (A) Representative histogram of the phenotype of Cellistem^TM^ assessed by flow cytometry analysis using different antibodies that include CD73, CD90, CD105, CD45, CD19, HLA-DR, CD34 and CD11b. Grey histogram represent the isotype control while red histogram represent the expression of the different mesodermal and non-mesodermal markers. (B) Differentiation potential into adipocytes, chondrocytes and osteoblast of Cellistem^TM^ was performed using specific staining techniques. For adipogenic differentiation, Oil-red staining was used to visualize lipid droplets, which were observed using phase contrast microscopy. For osteogenic differentiation, alizarin red staining was employed to visualize mineralization, also observed using phase contrast microscopy. Finally, safranin O staining was used to observe proteoglycans for chondrogenic differentiation, again using phase contrast microscopy. (C) TSP-2 secretion in the supernatants of 3 different batches of UC-MSC isolated from different donors was evaluated by ELISA. (D) Karyotype evaluation of Cellistem^TM^ .
