# Supplementary figures and images for: A Phase I Dose-Escalation Clinical Trial to Assess the Safety and Efficacy of Umbilical Cord-Derived Mesenchymal Stromal Cells in Knee Osteoarthritis
Source: Stem Cells Transl Med. 2024 Feb 16;13(3):193–203. doi: 10.1093/stcltm/szad088 (PMC10940813; doi:10.1093/stcltm/szad088)

A)

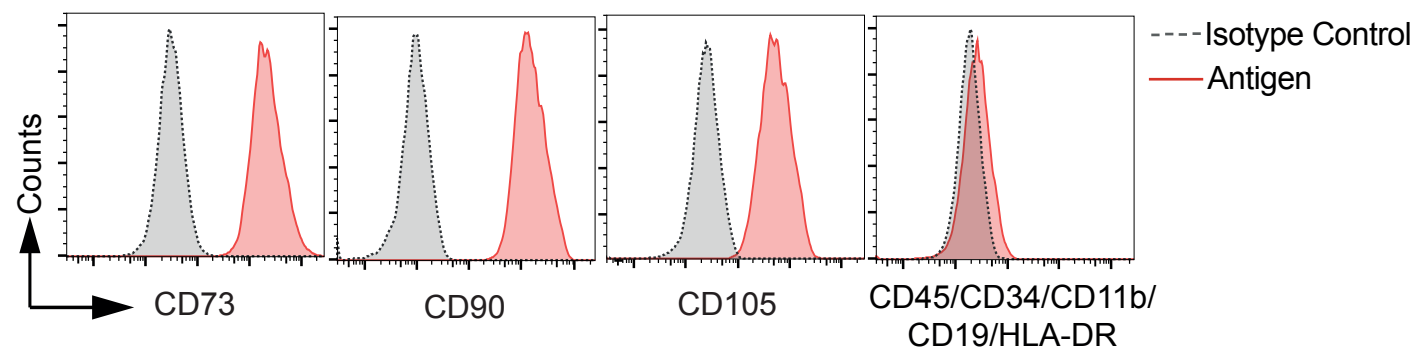

B)

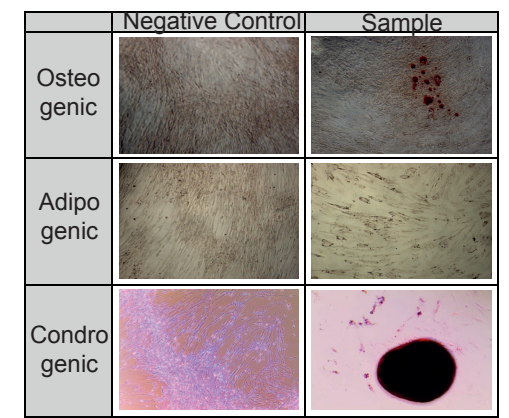

C)

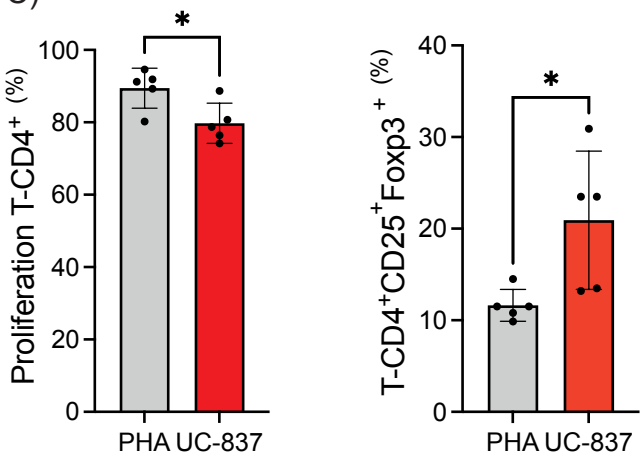

D)

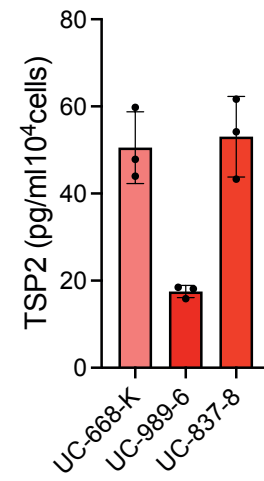

E)

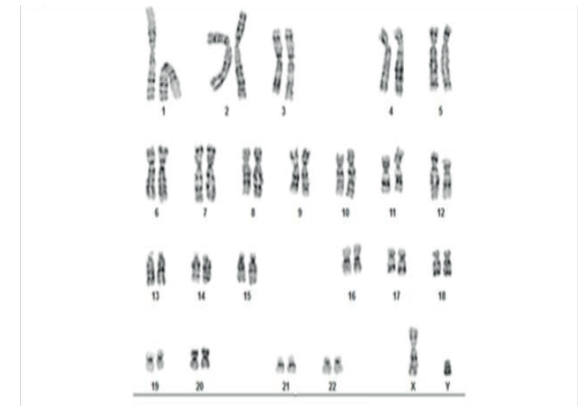

Supplement: szad088_suppl_Supplementary_Figure_1 [file szad088_suppl_supplementary_figure_1.zip › newfolder/Supp. Figure 1.pdf]
