# Supplementary material for: A Phase I Dose-Escalation Clinical Trial to Assess the Safety and Efficacy of Umbilical Cord-Derived Mesenchymal Stromal Cells in Knee Osteoarthritis
Source: Stem Cells Transl Med. 2024 Feb 16;13(3):193–203. doi: 10.1093/stcltm/szad088 (PMC10940813; doi:10.1093/stcltm/szad088)
Supplement: szad088_suppl_Supplementary_Table_1 [file szad088_suppl_supplementary_table_1.docx]

**Supplementary Table 1**: **Characteristics of pain related to MSC injection.**
